# Progress of Surgery

**Published:** 1900-10-27

**Authors:** 


### Progress of Surgery.

### SURGERY OF THE LIVER AND GALL-BLADDER.

**Neoplasms of the Liver** are rarely seen early enough to allow of their removal. The adenomata and a few carcinomatous deposits—either primary, or secondary by continuity of tissue alone—are the only malignant tumours ever likely to be considered operable. It is only in such cases in which there is distinct delimitation of the disease that we can hope to do anything of a radical nature, and it is also evident that as disease approaches the hilus it becomes more dangerous and less suitable to any operative treatment. Experience has shown that the cautery and ligature, alone or combined, are the safest and quickest means of controlling bleeding from hepatic tissue, and that pressure with packing is an useful adjuvant. The cauterised wound in the liver should be treated by packing, and the abdominal cavity protected from contamination by the same means. All means of combating the effects of hæmorrhage should be ready for immediate use, for it is never certain that we can avoid serious trouble in this regard. There is no prospect that any but growths n the superficial portions of the liver can be attacked successfully, even supposing that it were possible to make a diagnosis with reasonable certainty when the tumour lies very deep. Bile from healthy liver tissue will not hurt the peritoneum, but from inflamed and infected gall-ducts it will cause peritonitis.[1]

**Cholelithiasis.**—Richardson [2] says gall-stones must be removed as soon as diagnosed. The indications for operation consist in finding facetted stones in the stools, non-facetted stones so found contra-indicating operation. Repeated attacks with or without finding stones points to operation. Quiescence no more contra-indicates operation than it does in appendicitis. Repeated attacks of tenderness and persistent pain over the gall-bladder are among the most valuable indications. Kehr [3] has performed operations for gall-stones in 491 cases during the past ten years, the mortality being from 2 to 7 per cent. Some patients continue to complain of colicky pain after operation. These troubles, he thinks, are due to calculi left in the gall-bladder because they are not found during the operation, and are never caused by the formation of fresh calculi. Kehr therefore suggests the name of "spurious relapses" to the pains originating in the manner above described. Such spurious relapses may be produced, moreover, by adhesions between the gall-bladder and its surroundings. In order that calculi may not be left behind he recommends total extirpation of the gall-bladder, as in this way better results are obtained than by incision and drainage. By early operation perforation of the gall-bladder, peritonitis, cholangitis, and carcinomatous degeneration are avoided. Jöbker[4] approves of total extirpation, but Petersen[5] prefers drainage of the gall-bladder. Koerte[6] is of opinion that an operation should be performed only when the pain is severe and frequent. The operation itself might produce adhesions which are usually very painful. Gibson[7] believes that cholecystectomy, as a means of treating cholecystitis and cholelithiasis, is called for under the following conditions:—In all cases of cholecystitis, with or without gall-stones, acute or chronic, provided that the gall-bladder and gall-ducts can be properly explored, and that the conditions promise an easy removal of the gall-bladder. It is to be borne in mind that the more distended the gall-bladder, the more likely is its separation from the liver to be easy. The operation is also recommended even when its performance is difficult or possibly entails a slightly greater risk in a limited class of cases—that is, when it is the only satisfactory way to deal with the gall-bladder, and as a prophylactic measure against malignant disease in the presence of long-standing irritation. If these various limitations receive a strict interpretation, the number of cholecystectomies that are justified by the above indications will be comparatively restricted, and the operation will be done only under circumstances that permit of its greatest usefulness with a minimum of risk. Lejars[8] says there are three principal forms of calculous cholecystitis: (1) A large bladder, clear fluid, solitary calculus impacted in the neck of the bladder or in the cystic duct. (2) The bladder is filled with a great number of calculi, varying in size and form, sometimes hundreds in number. (3) Where the gall-bladder is retracted, thickened, and contains one large or a few small calculi. Operation is valuable in all these forms. Bolognesi[9] says that in cases of cholelithiasis without jaundice, diagnosis is difficult; but the author claims that there is in all cases of cholelithiasis increase in size of the liver, most marked during the crises, and lessening as these pass off, but persisting in some degree in all chronic cases of biliary calculus. The enlargement may not be found in front, but examination will then show it in the axillary line or posteriorly. He lays stress upon the value of this sign in diagnosis and in deciding upon surgical intervention. James Swain[10] records a case of cholecystotomy in which no gall-stones were present. The case proved to be one of simple hydrops of the gall-bladder caused by kinking of the cystic duct from adhesions between the colon and the gall-bladder. Battle[11] contributes three cases of choledochotomy, and Garrett[12] one of traumatic rupture of the common bile duct.

[1] Med. Rec., March 24, 1900, p. 502. [2] Ibid., June 9, 1900, p. 993. [3] Lancet, May 12, 1900, p. 1402. [4] Ibid. [5] Ibid. [6] Ibid. [7] Med. Rec., June 9, 1900, p. 977. [8] Amer. Jour. Med. Sci., March, 1900, p. 350. [9] Med. Chron., May, 1900, p. 133. [10] Brit. Med. Journ., June 2, 1900, p. 1329. [11] Lancet, May 19, 1900, p. 1442. [12] Annals of Surg., Feb. 1900, p. 227.

